# Therapeutic efficacy of dose-reduced adjuvant chemotherapy with S-1 in patients with pancreatic cancer: a retrospective study

**DOI:** 10.1186/s12885-022-10116-2

**Published:** 2022-09-30

**Authors:** Kazuki Kobayashi, Takahiro Einama, Yasuhiro Takihata, Naoto Yonamine, Ibuki Fujinuma, Takazumi Tsunenari, Keita Kouzu, Akiko Nakazawa, Toshimitsu Iwasaki, Hideki Ueno, Yoji Kishi

**Affiliations:** Department of Surgery, National Defense Medical College, 3-2 Namiki, Tokorozawa, Saitama 359-8513 Japan

**Keywords:** Pancreatic cancer, S-1, Adjuvant chemotherapy, Total dose intensity, Relative dose intensity

## Abstract

**Background:**

S-1 adjuvant chemotherapy is the standard treatment in Asia for resectable pancreatic ductal adenocarcinoma. The relative dose intensity of adjuvant chemotherapy influences survival in pancreatic cancer but does not precisely reflect treatment schedule modifications. We investigated the effects of total dose intensity of S-1 adjuvant chemotherapy on the survival of patients with pancreatic cancer and the permissible dose reduction.

**Methods:**

Patients who underwent surgical resection during 2011–2019 for pancreatic cancer were selected. We determined the total dose intensity cut-off value that predicted tumor recurrence within 2 years postoperatively using receiver operating characteristic curves and compared the outcomes between the high and low total dose intensity groups.

**Results:**

Patients with total dose intensity ≥ 62.5% (*n* = 53) showed significantly better overall survival than those with total dose intensity < 62.5% (*n* = 16) (median survival time: 53.3 vs. 20.2 months, *P* < 0.001). The median survival of patients without adjuvant chemotherapy (total dose intensity = 0, *n* = 28) was 24.8 months. Univariate analysis identified lymphatic involvement (*P* = 0.035), lymph node metastasis (*P* = 0.034), and total dose intensity (*P* < 0.001) as factors affecting survival. On multivariate analysis, total dose intensity (*P* < 0.001) was an independent predictor of worse survival.

**Conclusions:**

Maintaining a total dose intensity of at least 60% in S-1 adjuvant chemotherapy seems important to achieve a long postoperative survival in patients with pancreatic cancer.

**Supplementary Information:**

The online version contains supplementary material available at 10.1186/s12885-022-10116-2.

## Introduction

Despite the recent improvements in surgical techniques [1], postoperative management [2], neoadjuvant chemotherapy [3, 4], and adjuvant chemotherapy (AC) [4], pancreatic cancer remains one of the most fatal malignancies. The European Study Group for Pancreatic Cancer (ESPAC) demonstrated that AC (gemcitabine or fluorouracil plus folic acid) improves the prognosis of pancreatic ductal adenocarcinoma (PDAC) [5]. In Japan, following the results of the Japan Adjuvant Study Group of Pancreatic Cancer (JASPAC) 01, AC with S-1 has become the standard treatment for resectable PDAC [6].

Previous reports suggested that the completion of planned AC is an important prognostic factor [7, 8]. However, these studies only evaluated whether or not AC was completed, while the impact of dosage or duration of treatment on the prognosis were not specifically assessed. Although the study which enrolled the patients in ESPAC-3 suggested that the prognosis was influenced more by the duration of chemotherapy at initiation [9], this study did not assess the dose intensity. Regarding dosage and prognosis, previous studies have suggested that decreased relative dose intensity (RDI) is associated with a poor prognosis [8, 10]. On the other hand, RDI does not precisely reflect dose reduction, treatment schedule modifications, or prolongation due to adverse effects, which are often experienced by patients with pancreatic cancer who receive S-1 AC.

The impact of AC dose on prognosis has remained unknown, and a useful evaluation index that can reflect the limitations inherent in RDI has not been established. We hypothesized that total dose intensity (TDI), which accounts for the actual administered amount, would more accurately reflect the patient’s prognosis. Therefore, we investigated the influence of the TDI of S-1 AC on the survival of patients with pancreatic cancer.

## Methods

### Ethics statement

The present study was approved by the Institutional Review Board of the National Defense Medical College (Approval No. 4115). All participants provided informed consent.

### Patients and study design

We selected patients who underwent upfront macroscopically curative resection for pancreatic cancer at the Department of Surgery, National Defense Medical College Hospital, Japan, between November 2011 and March 2019. Patients who received preoperative chemotherapy and those who initiated postoperative AC with other regimens than S-1 monotherapy were also excluded. The patients who could not tolerate adjuvant S-1 chemotherapy were not switched to the other regimens. All patients had a histo-pathologically confirmed diagnosis of pancreatic cancer.

We calculated the TDI in all patients and the patients were classified into three groups by TDI. The effects of the reduced administered dose of S-1 were examined by comparing the relapse free survival (RFS) and overall survival (OS) of the patients. Furthermore, the survival time after recurrence was also examined. The median RFS, OS, survival time after recurrence, and 5-year OS rates are presented. The data on tumor markers including serum CA19-9 values after biliary drainage, as well as the serum total bilirubin < 5 mg/dL were collected on patients with obstructive jaundice initially.

### Surgical procedures

Operative methods were determined based on tumor location. All patients underwent regional lymph node dissection. The pathologic stage was assessed according to the TNM classification system of the Union for International Cancer Control (UICC), 8^th^ edition.

### Chemotherapy regimens

The AC regimen used for each patient was S-1 monotherapy. The regimen consisted of S-1 80–120 mg/day, depending on body surface area (< 1.25 m^2^: 80 mg/day, 1.25–1.50 m^2^: 100 mg/day, > 1.50 m^2^: 120 mg/day), administered twice a day for 4 weeks followed by withdrawal for 2 weeks and repeated every 6 weeks for 4 courses, or administered twice a day for 2 weeks followed by withdrawal for 1 week and repeated every 3 weeks for 8 cycles. AC was planned to be administered for 24 weeks (total period of administration of 112 days) and was initiated as soon as possible after the patient’s physical condition recovered after surgery.

### TDI

TDI was calculated for each patient according to the following formula:

$$\text{TDI} = \text{(real dose}\times\text{real administration days)}/\text{(ideal dose}\times\text{ideal administration days)}\times\text{100.}$$

For example, if a person with a body surface area of 1.5 m^2^ was administered 100 mg for 100 days, the TDI was (100 × 100)/(120 × 112) × 100 = 74.4%.

### Cut-off value to predict recurrence after surgery

We constructed a receiver operating characteristic (ROC) curve using logistic regression to determine the cut-off value of TDI that predicted tumor recurrence within 2 years after surgery. Because most cases relaps within 2 years after surgery [11]. The Youden index [= sensitivity—(1—specificity)] was calculated, and the TDI with the highest Youden index was selected as the optimal cut-off value.

### The reasons of failure to complete AC

The reasons of failure to complete AC were classified as adverse events, tumor recurrence, death of any cause, and others. The adverse events of AC were evaluated using the Common Terminology Criteria for Adverse Events (CTCAEv5.0) [12]. Complications of grade 3 or higher were considered major morbidities.

### Postoperative follow-up

After pancreatectomy, each patient was followed up at outpatient clinics. Tumor markers and computed tomography (CT) were evaluated every two months and three months, respectively, during AC, and every three months and six months, respectively after AC, until 5 years after surgery. After that, tumor makers and CT were evaluated every 6 months. Diagnosis of recurrence was made based on CT findings.

### Statistical analysis

Continuous variables were expressed as medians and ranges. The correlation of numerical and categorical variables between high TDI, low TDI, and no AC groups were evaluated using one way analysis of variance (ANOVA) and Pearson’s χ^2^ test, respectively. RFS, OS, and survival after recurrence were calculated using the Kaplan–Meier method, and differences in survival were compared using the log-rank test. For the estimation of OS, patients remaining alive were censored at the date of the last follow up. RFS was measured from the date of resection to the date of death from any cause or date of recurrence or metastasis. Only the patients remaining alive without recurrence were censored. Univariate and multivariate analyses to determine the predictors of worse OS were performed using the Cox proportional hazards model. Variables with *P* < 0.05, in the univariate analyses, were entered into the multivariate analyses. All statistical analyses were performed using EZR (Saitama Medical Center, Jichi Medical University, Saitama, Japan), which is a graphical user interface for R (The R Foundation for Statistical Computing, Vienna, Austria) and, more precisely, a modified version of R commander designed to add the statistical functions frequently used in biostatistics.

## Results

### Patient selection

During the study period, 138 patients received surgical resection for the preoperative diagnosis of pancreatic cancer. Among them, 14 patients who had an initial diagnosis of unresectable disease; nine who patients had a pathologic diagnosis of non-invasive carcinoma, 14 patients who had received pre-operative chemotherapy, and four patients who had started AC with other regimens than S-1 were excluded. The remaining 97 patients were included in this study. From this group, 69 patients received AC with S-1, while the other 28 did not. The median age was 71 years (range, 50–92 years). Sixty-two patients (64%) were men. Sixty-four (66%), 28 (29%), and five (5%) patients underwent pancreaticoduodenectomy, distal pancreatectomy, and total pancreatectomy, respectively. Sixty-nine patients (70%) received S-1 AC, and 28 patients (41%) completed the planned 24-week treatment. Twenty-eight patients did not receive AC; the reasons for this were: recurrence (four patients, 14%), old age (seven patients, 25%), poor performance status (four patients, 14%), and others (13 patients, 46%).

### Determination of the cut-off value of TDI

**Fig. 1:**
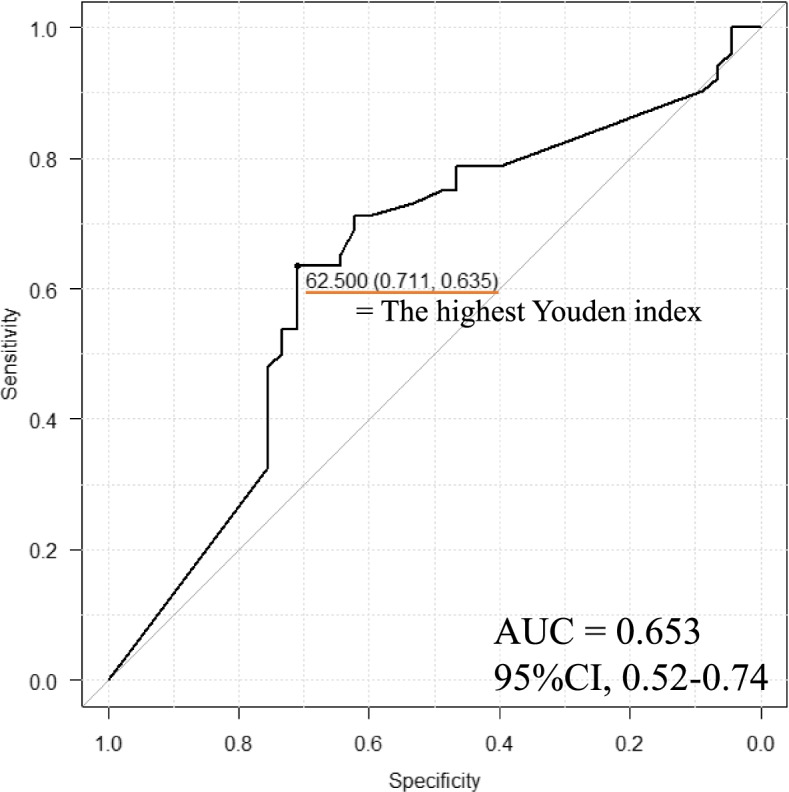
Receiver operating characteristic (ROC) curve to determine the cut-off value. The value is 62.5%. The area under the curve (AUC) is 0.653 and the 95% confidence interval (CI) is 0.52–0.77

Figure 1 shows the ROC curve used to determine the appropriate cut-off value for the TDI. The cut-off was determined as 62.5% [area under the curve (AUC) = 0.653; 95% confidence interval (CI): 0.52–0.74]. Table 1 shows a comparison of the pre-operative profiles, surgical procedures, and postoperative clinical and pathologic outcomes between patients with high TDI, low TDI, and no AC.

**Table 1:** Patient characteristics

	**Total** **(*n* = 97)**	**High TDI** **(*n* = 53)**	**Low TDI** **(*n* = 16)**	**AC(-)** **(*n* = 28)**	***P* value**	***P* value** **(high vs. low)**
Sex (male/female)	62/35	32/21	8/8	22/6	0.122	0.468
Age, years	71 (50–92)	70 (50–85)	68.5 (54–79)	77.5 (59–92)	< 0.001	0.863
Preoperative CEA	2.6 (0.6–45.2)	2.4 (0.6–45.2)	4.0 (1.5–24.6)	2.7 (1–30.6)	0.507	0.344
Preoperative CA19-9	135 (0.4–4510)	108.4 (0.7–4510)	85.0 (0.4–1511)	198 (20–2650)	0.955	0.891
Operative method					0.133^a^	0.044^a^
-PD	64	32	14	18		
-DP	28	19	1	8		
-TP	5	2	1	2		
Pathological diagnosis (IDC/inv. IPMC)	68/29	38/15	13/3	17/11	0.179	0.163
Pathological T factor (1/2/3/4)	12/67/12/6	7/35/8/3	1/12/1/2	4/20/3/1	0.324	0.586
ly (0/1/2/3)	12/64/12/9	7/39/5/2	0/9/3/4	5/16/4/3	0.204	0.164
v (0/1/2/3)	9/31/43/14	1/14/28/10	0/2/10/4	8/15/5/0	0.665	0.586
UICC stage					0.102^b^	0.586^b^
-IA, IB	2	1	0	1		
-IIA	14	6	1	7		
-IIB	41	25	5	11		
-III	37	19	10	8		
-IV	3	2	0	1		
Pathological lymph node metastasis (positive/negative)	80/17	45/8	15/1	20/8	0.139	0.365
Residual tumor (R0/R1)	86/11	48/5	13/3	25/3	0.592	0.315
Time to start AC after surgery, days	53 (24–399)	48 (27–399)	78.5 (24–110)	none	0.436	0.436

*TDI* Total dose intensity, *PD* Pancreatoduodenectomy, *DP* Distal pancreatectomy, *TP* Total pancreatectomy, *ly* Lymphatic permeation, *v* Blood vessel invasion, *AC* Adjuvant chemotherapy, *IDC* Invasive ductal carcinoma, *inv. IPMC* Invasive intraductal papillary mucinous carcinoma

^a^PD vs. DP and TP, ^b^Stage ≥ IIB vs. < IIB

### Comparison of profiles according to TDI

Patients were divided into a high-TDI group (TDI ≥ 62.5%; *n* = 53), low-TDI group (TDI < 62.5%; *n* = 16), and AC(-) group (TDI = 0%; *n* = 28). The background characteristics of the two groups are shown in Table 1. Age differed significantly between the three groups, and median age of the patients in the AC(-) group was the highest (the median age of the high-TDI, low-TDI, and AC(-) groups was 70, 68.5, and 77.5 years, respectively; *P* < 0.001). However, there were no significant differences in perioperative profiles between the high- and low-TDI groups.

### Reasons for incomplete adjuvant chemotherapy

In total, 24 patients in the high-TDI group did not complete AC. Table 2 summarizes the reasons for cessation. The main reasons why AC could not be completed in the high- and low-TDI groups were: recurrence (high-TDI, 11 patients, 46%; low-TDI, 5 patients, 6%; *P* = 0.50) and adverse events (high-TDI, 4 patients, 17%; low-TDI, ten patients, 63%; *P* = 0.003). Adverse events included nausea (3 patients, 21%); diarrhea (1 patient, 7%); heart failure (1 patient, 7%); and interstitial pneumonia (1 patient, 7%). All the adverse events were CTCAE Grade 3.

**Table 2:** The causes of failure to complete adjuvant chemotherapy

	High TDR (*n* = 24)	Low TDR (*n* = 16)	*P* value
Recurrence	10 (42%)	5 (31%)	0.504
Adverse event (CTCAE Grade 3 or 4)	4 (16%)	10 (63%)	0.003
-Nausea	0(0%)	3 (30%)	
-Diarrhea	1 (25%)	0 (0%)	
-Heart failure	1 (25%)	0 (0%)	
-Interstitial pneumonia	0 (0%)	1 (10%)	
-Liver dysfunction	0 (0%)	2 (20%)	
-Others	2 (50%)	4 (40%)	
Death	1 (5%)	0 (0%)	0.408
Others^a^	9 (38%)	1 (6%)	0.013

*TDI* Total dose intensity, *CTCAE* Common Terminology Criteria for Adverse Events

^a^Low-performance status or the patient’s decision to not continue treatment were included

### Survival outcomes

The median follow-up time was 31.7 months. Totally, 27 patients were censored including seven without tumor relapse.

**Fig. 2:**
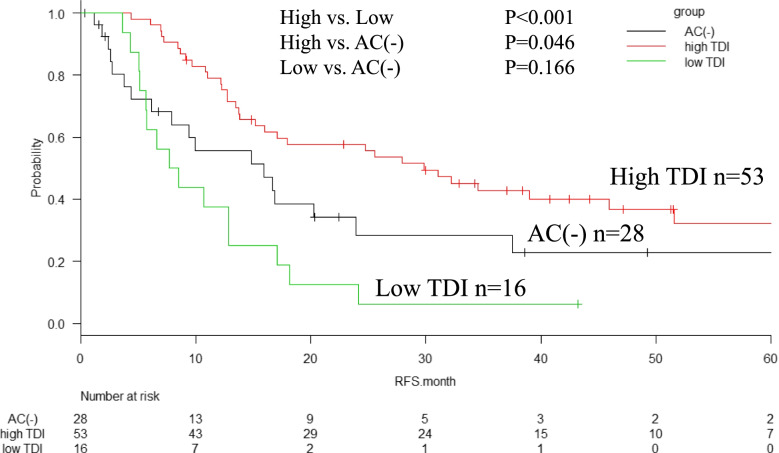
Relapse free survival (RFS) of patients according to the total dose ratio (TDI) of adjuvant chemotherapy (AC). The high-TDI group shows significantly better RFS than the low-TDI group and AC(-) group (median, 30 months vs. 8 months, *P* < 0.001) and AC (-) group (median, 16 months, vs. high-TDI, *P* = 0.046). There are no significant difference between low-TDI group and AC(-)group (*P* = 0.166)

**Fig. 3:**
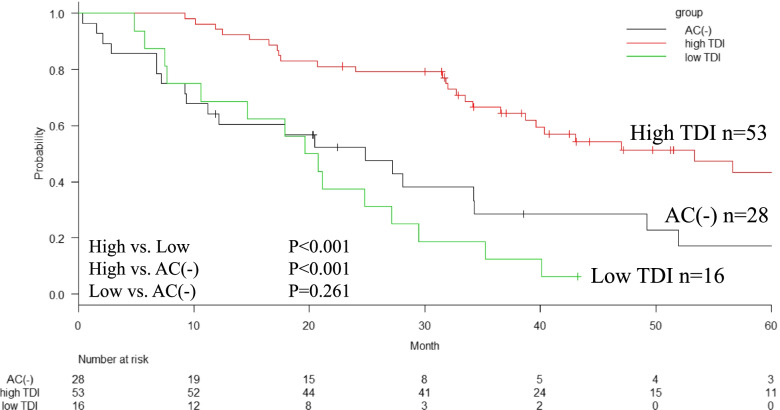
Overall survival (OS) of patients according to the total dose ratio (TDI) of adjuvant chemotherapy (AC). The high-TDI group shows significantly better OS than the low-TDI group and AC(-) group (median, 53 months vs. 20 months, *P* < 0.001) and AC (-) group (median, 25 months, vs. high-TDI, *P* < 0.001)

**Fig. 4:**
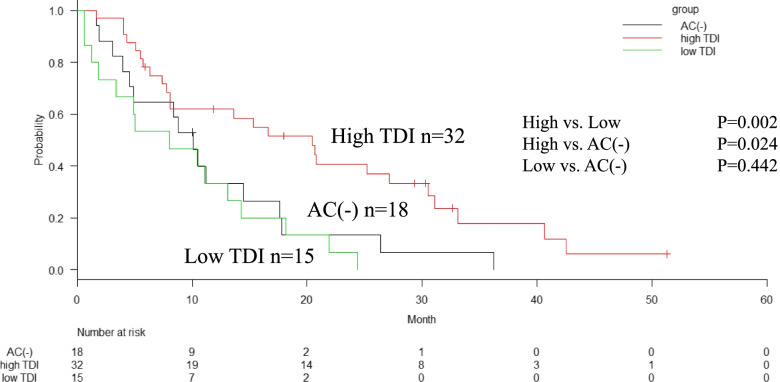
Survival after recurrence of patients according to the total dose ratio (TDI) of adjuvant chemotherapy (AC). The high-TDI group shows significantly better survival than low-TDI group (median, 20 months vs. 9 months, *P* = 0.002) and AC(-) group (median, 10 months, vs. high-TDI, *P* = 0.024)

The median RFS in the high-TDI, low-TDI, and AC(-) groups were 30, 8, and 16 months, respectively with significant difference (*P* < 0.001) (Fig. 2). The median survival times (MST) in the high-TDI, low-TDI, and AC(-) groups were 53, 20, and 25 months, respectively. The 5-year OS rates in the high-TDI, low-TDI, and AC(-) groups were 43%, unavailable, and 17%, respectively (*P* < 0.001) (Fig. 3). The median survival time after recurrence in the high-TDI, low-TDI, and AC(-) groups were 20, 8, and 10 months, respectively (*P* = 0.003) (Fig. 4).

### Univariate and multivariate analyses for the predictors of poor OS

We determined the carbohydrate antigen 19–9 (CA19-9) cut-off value as 338.45 U/ml from a previous study [13]. Univariate analyses showed that lymphatic permeation (hazard ratio [HR] = 3.50; 95% confidence interval [CI], 1.09–11.2; *P* = 0.035), lymph node metastasis (HR = 2.69; 95% CI: 1.07–6.75; *P* = 0.034), and TDI (HR = 3.10; 95% CI, 1.86–5.15; *P* < 0.001) were significant predictors of worse OS. Multivariate analysis of these variables (with *P* < 0.050) showed that TDI (HR = 3.38; 95% CI, 2.01–5.70; *P* < 0.001) was an independent factor affecting survival (Table 3).

**Table 3:** Univariate and multivariate logistic regression analysis of overall survival

Parameter	Univariate		Multivariate	
	HR (95% CI)	*P* value	HR (95% CI)	*P* value
Sex (men vs. women)	1.02 (0.61–1.72)	0.912		
Age (< 70 vs. ≥ 70 years)	1.19 (0.72–1.95)	0.488		
Preoperative CA19-9 (< 338.45 vs. ≥ 338.45)	1.04 (0.60–1.80)	0.871		
Tissue size (3, 4 vs. 1, 2)	1.12 (0.56–2.22)	0.754		
ly (positive vs. negative)	3.50 (1.09–11.2)	0.035^a^	2.27 (0.56–9.09)	0.246
v (positive vs. negative)	1.36 (0.96–1.93)	0.082		
Pathological T factor (≥ 3 vs. < 3)	1.09 (0.15–7.95)	0.928		
Lymph node metastasis (positive vs. negative)	2.69 (1.07–6.75)	0.034^a^	2.03 (0.74–5.52)	0.164
Residual tumor (≥ 1 vs. 0)	1.94 (0.94–4.01)	0.073		
Time to start AC (≥ 42 vs. < 42 days)	1.21 (0.65–2.22)	0.527		
TDI (< 62.5% vs. ≥ 62.562.5)	3.10 (1.86–5.15)	< 0.001^a^	3.38 (2.01–5.70)	< 0.001^a^

*HR* Hazard ratio, *ly* Lymphatic permeation, *v* Blood vessel invasion, *AC* Adjuvant chemotherapy, *TDI* Total dose intensity

^a^Statistically significant

## Discussion

The present study suggested that a long OS was achieved only when a high TDI of at least 60% was maintained in postoperative AC with S-1. Dose reduction or schedule modifications could be acceptable if at least 60% of the total dose was administered. In addition, we found was no significant difference between patients who received < 60% of the total dose and the AC(-) group.

As the reason for failure to complete AC, the occurrence of adverse events was predominant in the low-TDI group. Therefore, tolerability of S-1 AC is essential. A previous study also showed that patients who completed the planned S-1 treatment had longer survival than those who discontinued treatment [9, 10]. JASPAC 01 revealed that 72% of patients completed planned chemotherapy cycles and 41% of patients received dose reduction [6]. The causes of failure to complete the planned cycles were grade 3–4 adverse events (according to the criteria of the Common Terminology Criteria for Adverse Events [12]), such as anemia (14%), neutropenia (13%), and recurrence (5%). However, existing guidelines often do not indicate how much dose reduction is permissible. It is anticipated that the findings of this study will help develop dose-reduction criteria in the future.

To the best of our knowledge, there are no reports on the appropriate dose of adjuvant S-1 monotherapy for pancreatic cancer. We constructed an ROC curve and determined the cut-off value of TDI as 62.5%. Patients who received S-1 adjuvant chemotherapy often experienced adverse events. If grade 3–4 adverse events were observed, dose reduction or treatment schedule modification was required. Previous reports have shown that the rate of patients who failed to complete S-1 adjuvant chemotherapy was 28%–32% [6, 14]. By reducing the dose in these patients, adverse events could be minimized. As a result, a sufficient dose might be administered to maintain at least the required TDI in order to improve OS.

Three possible causes for better OS in the high-TDI group than in the low-TDI group are considered. First, this study included of the patients with recurrence during AC. Although the incidence of recurrence during the adjuvant S-1 treatment was comparable between patients in the high-TDI group and the low-TDI group, recurrence occurred earlier in the low-TDI group. High frequency of early recurrence might have had a considerable influence on the prognosis. However, in this study, we did not exclude the patients with early recurrence during AC, to minimize the effect of selection bias. Second, the survival time after recurrence was significantly longer in the high-TDI group. The patients in the high-TDI group might be highly tolerant to chemotherapy. These patients might have been more likely to receive sufficient dose of systemic chemotherapy even after the recurrence, although the details of treatment following recurrence were not evaluated in this study. Third, the number of patients, especially the low-TDI group patients was small. The small sample size might have influenced the prognostic outcome. We are currently planning a multi-institutional study to investigate a more feasible cut-off of TDI to obtain a long survival.

Yabusaki et al. reported about the RDI of S-1 adjuvant chemotherapy [10]. RDI is often considered as an important prognostic factor for determining sufficient dosage and drug administration [15]. These reports revealed that low RDIs were associated with a poor prognosis. However, with RDI, it is difficult to precisely evaluate the optimal administration period for patients who resumed the treatment after transient withdrawal and whether it is feasible to wrap up with AC without extending the administration period in the cases with dose reduction. Since TDI is an evaluation index that reflects only the number of days and the amount of administration, it could solve these problems concerning the administration period. In addition, TDI is easier to calculate than RDI.

ESPAC-3 suggests the possibility that the administration period affects prognosis [9]. We had initially evaluated the prognostic effect focusing only on the duration of the S-1 AC. We calculated the ratio of the ideal and actual administration periods similar to that of TDI and constructed an ROC curve using a logistic regression model to determine the cut-off value of the duration ratio (DR) that predicted tumor recurrence within 2 years after surgery. The cut-off value was 0.851 (AUC, 0.653; 95% CI, 0.541–0.766) (supplemental Fig. 1). The long duration (DR(L)), short duration (DR(S)), and AC(-) groups were defined as DR ≥ 0.851, DR < 0.851, and DR = 0, respectively. Significant differences in RFS and OS were observed among the three groups (*P* < 0.001, *P* < 0.001, respectively) (supplemental Figs. 2 and 3). These results suggest that the duration affected the OS, similar to that of ESPAC-3. Although duration is a simple evaluation index, the results of this study using TDI suggest that S-1 as an AC, was effective up to the two-step reduction. Therefore, the TDI is also a simple and clinically useful index.

There are several limitations to our study. First, due to the low AUC, the validity of the TDI cut-off is controversial. However, a TDI of < 62.5% was an independent prognostic factor. Second, this was a retrospective, single-institution study with a limited number of patients. The possibility of overfitting due to small sample size exists. Thus, it is important to validate our results in another cohort or multicenter study. Third, whether it was feasible to include the patients with tumor recurrence during AC was concerned. Finally, our data included only patients who underwent upfront surgery and postoperative adjuvant chemotherapy. Whether the same results can be obtained in patients with pancreatic cancer who have received neoadjuvant chemotherapy needs to be investigated.

## Conclusion

The maintenance of TDI may be important for improving OS in patients with pancreatic cancer who have undergone curative resection with S-1 adjuvant chemotherapy. Our results showed that a low TDI was associated with a poorer prognosis, with a cut-off value of 62.5% showing the best predictive value.

## Supplementary Information

**Additional file 1.**

## Data Availability

The datasets used and/or analyzed during the current study available from the corresponding author on　reasonable request.

## Abbreviations

AC: Adjuvant chemotherapy
AUC: Area under the curve
CI: Confidence interval
ESPAC: European Study Group for Pancreatic Cancer
HR: Hazard ratio
JASPAC: Japan Adjuvant Study Group of Pancreatic Cancer
OS: Overall survival
PDAC: Pancreatic ductal adenocarcinoma
RDI: Relative dose intensity
TDI: Total dose intensity
