# Supplementary material for: Therapeutic efficacy of dose-reduced adjuvant chemotherapy with S-1 in patients with pancreatic cancer: a retrospective study
Source: BMC Cancer. 2022 Sep 30;22:1028. doi: 10.1186/s12885-022-10116-2 (PMC9524130; doi:10.1186/s12885-022-10116-2)
Supplement: Supplementary file 1 — Additional file 1. [file 12885_2022_10116_MOESM1_ESM.pptx]

## Slide 1
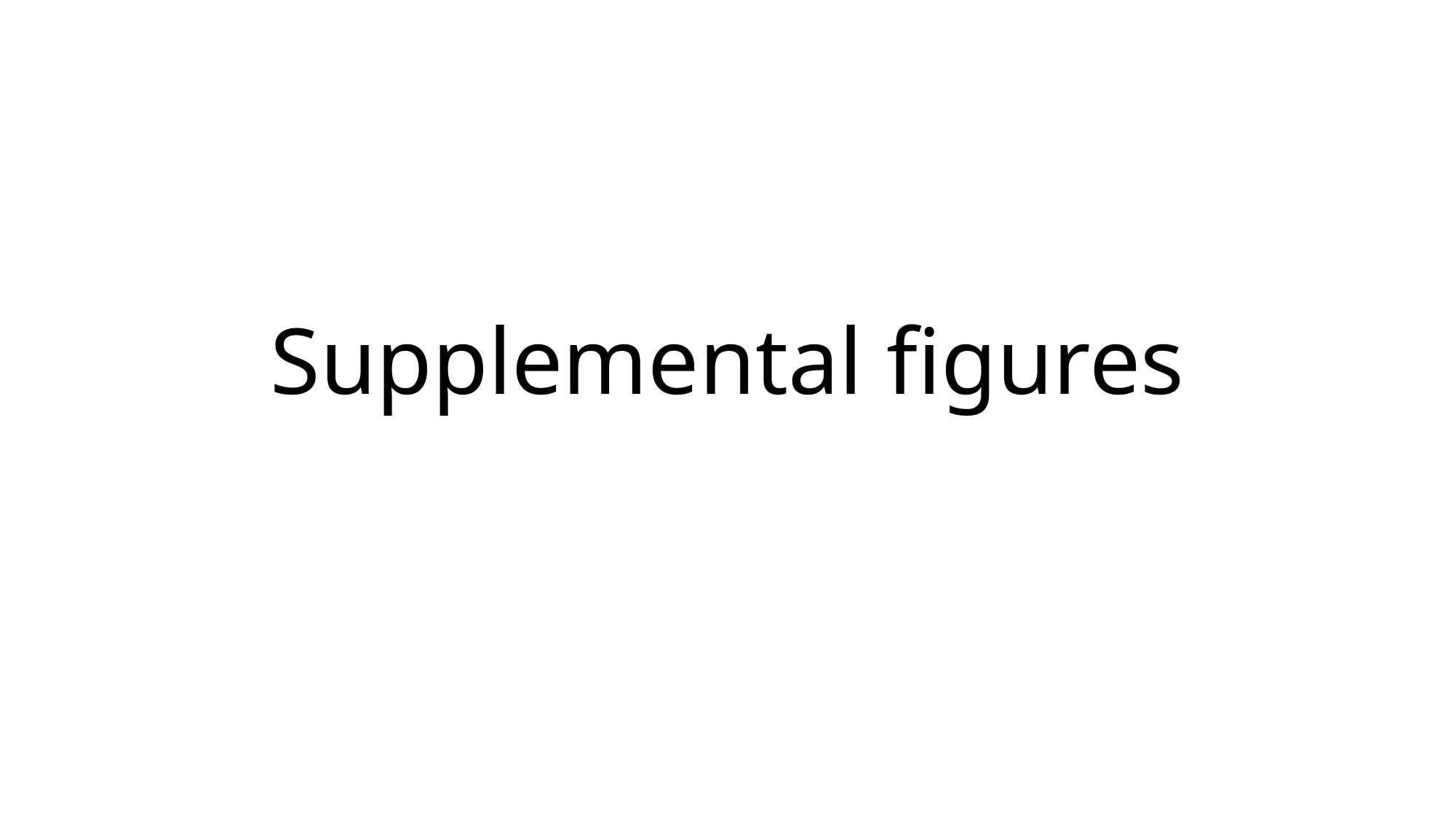

# Supplemental figures

## Slide 2
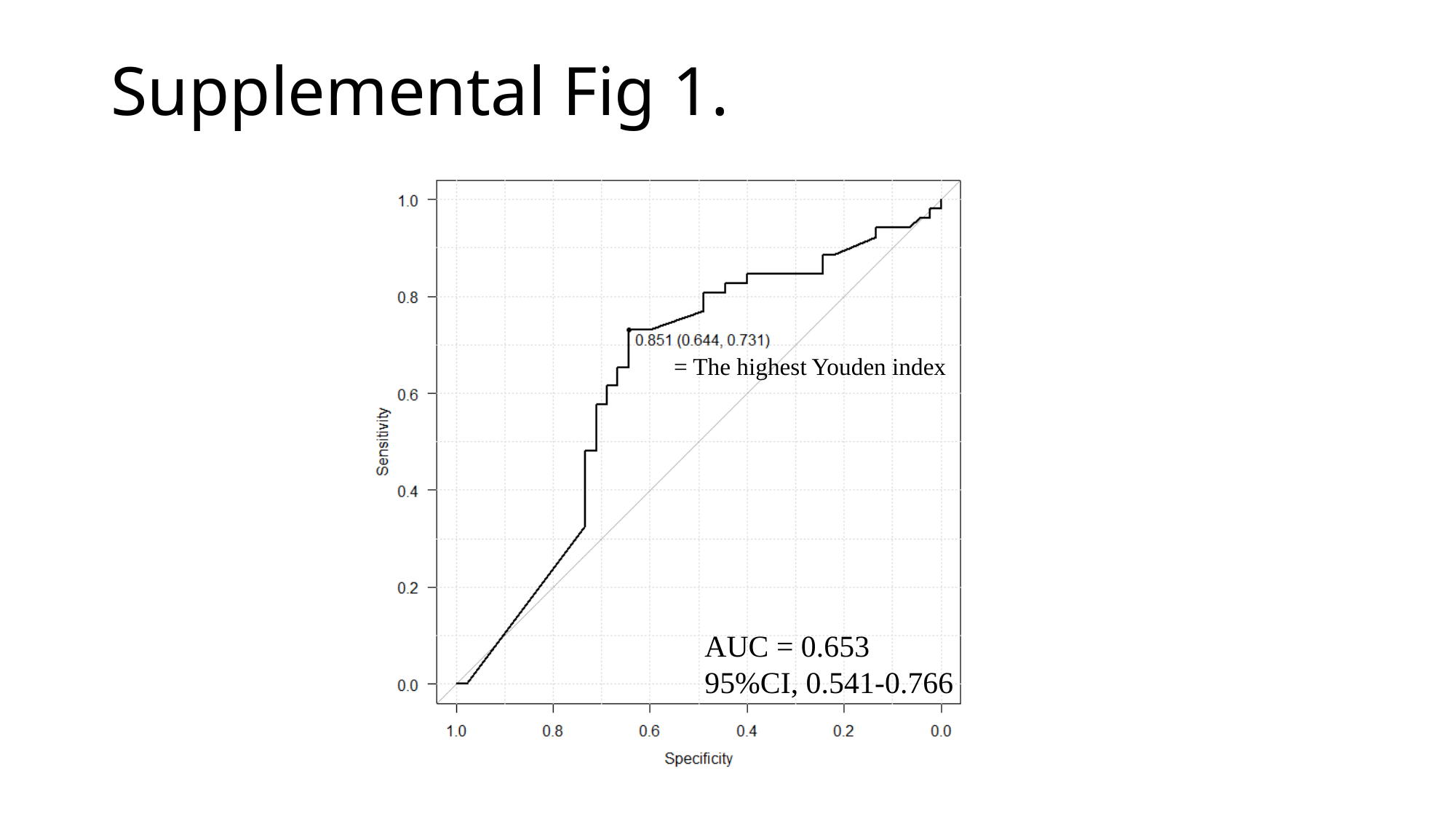

# Supplemental Fig 1.
= The highest Youden index
AUC = 0.653
95%CI, 0.541-0.766

## Slide 3
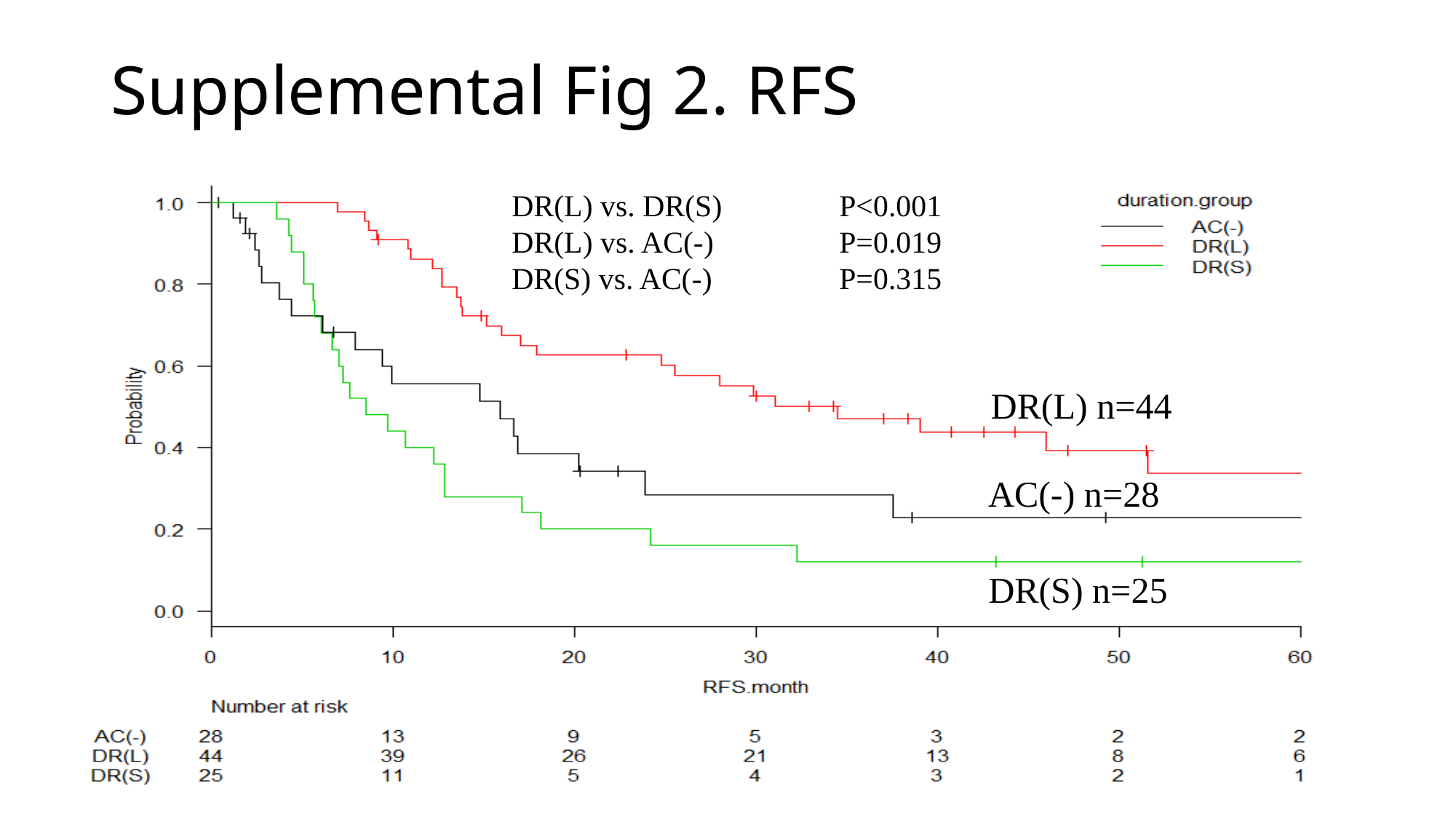

# Supplemental Fig 2. RFS
DR(L) vs. DR(S)		P<0.001
DR(L) vs. AC(-)		P=0.019
DR(S) vs. AC(-)		P=0.315
DR(L) n=44
AC(-) n=28
DR(S) n=25

## Slide 4
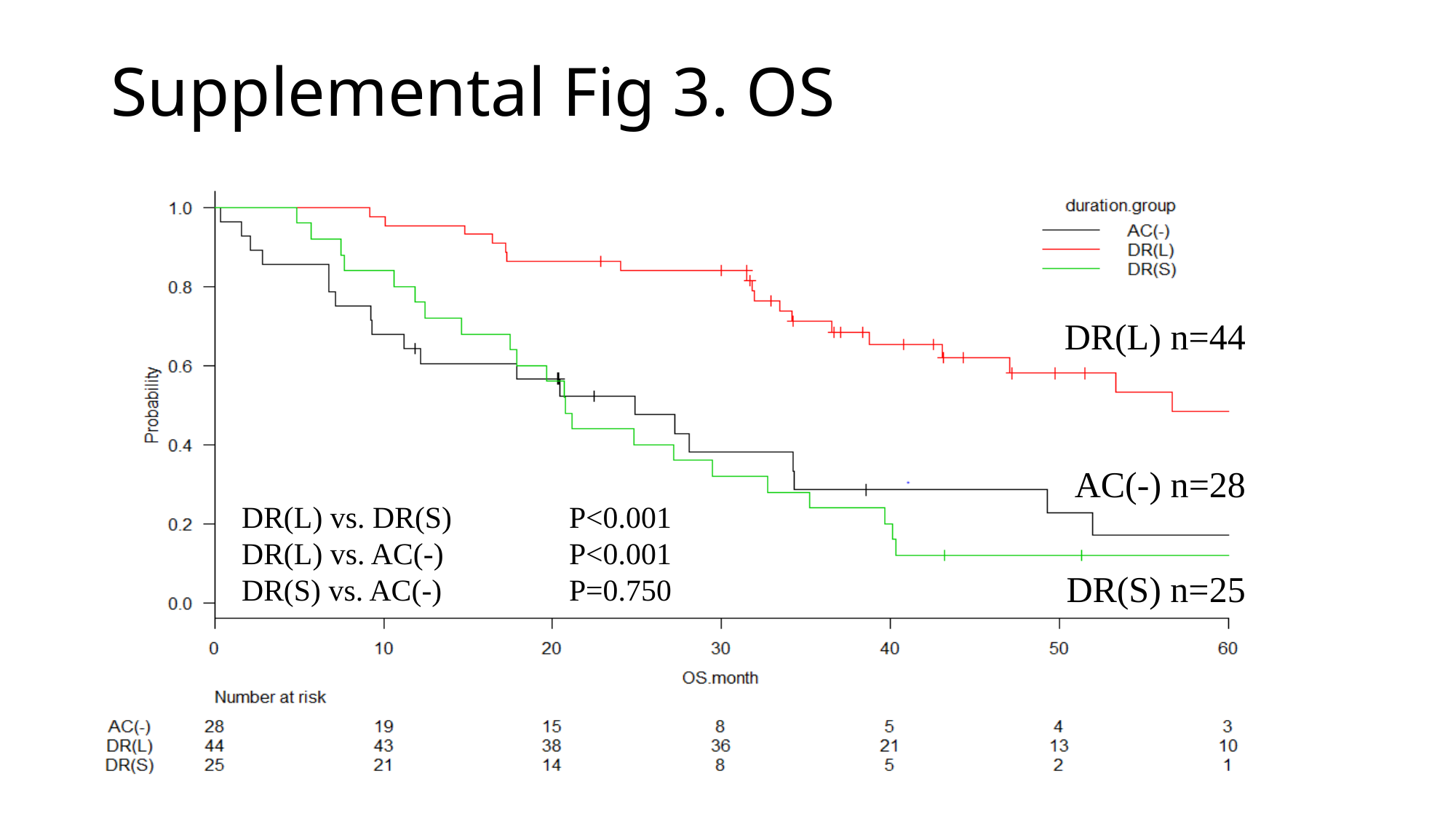

# Supplemental Fig 3. OS
DR(L) n=44
AC(-) n=28
DR(L) vs. DR(S)		P<0.001
DR(L) vs. AC(-)		P<0.001
DR(S) vs. AC(-)		P=0.750
DR(S) n=25
